# Impact of inhaled nitric oxide therapy in patients with cardiogenic shock treated with veno-arterial extracorporeal membrane oxygenation combined with Impella: a retrospective cohort study

**DOI:** 10.1186/s40560-024-00761-z

**Published:** 2024-11-18

**Authors:** Nobuhiro Yamada, Masafumi Ueno, Kyohei Onishi, Keishiro Sugimoto, Kazuyoshi Kakehi, Kosuke Fujita, Koichiro Matsumura, Gaku Nakazawa

**Affiliations:** Division of Cardiology, Department of Medicine, Faculty of Medicine, Kindai University, 377-2, Ohno-Higashi, Osakasayama, Osaka 589-8511 Japan

**Keywords:** Inhaled nitric oxide, Impella, Veno-arterial extracorporeal membrane oxygenation, Cardiogenic shock, Intensive care, Right ventricular function

## Abstract

**Background:**

The mortality rate of patients with cardiogenic shock (CS) requiring veno-arterial extracorporeal membrane oxygenation (VA-ECMO) combined with Impella (ECPELLA) support remains high. Inhaled nitric oxide (iNO) improves right ventricular (RV) function, resulting in increased Impella flow, which may facilitate early withdrawal of VA-ECMO and improve survival. This study investigated the prognostic impact of iNO therapy in ECPELLA patients.

**Methods:**

We retrospectively analyzed the data of consecutive patients with CS supported by ECPELLA from September 2019 to March 2024 at our hospital. Changes in pulmonary artery pulsatility index (PAPi) and Impella flow over time were evaluated, and VA-ECMO withdrawal rate, time to withdrawal, and 30-day survival were compared between ECPELLA patients with and without iNO therapy.

**Results:**

Of the 48 ECPELLA patients, 25 were treated with iNO. There were no significant differences between the groups in baseline characteristics or lactate levels at mechanical circulatory support induction. Patients with iNO therapy demonstrated significant improvements in the PAPi over time and a trend toward increased Impella flow, as well as a significantly higher VA-ECMO withdrawal rate (88% vs. 48%, P = 0.002) and a shorter time to VA-ECMO withdrawal (5 [3–6] days vs. 7 [6–13] days, P = 0.0008) than those without iNO therapy. Kaplan–Meier analysis demonstrated that the 30-day survival rate was significantly higher in patients with iNO than in those without (76% vs. 26%, P = 0.0002).

**Conclusions:**

iNO therapy in patients with CS requiring ECPELLA was associated with short-term prognosis by improving RV function and facilitating weaning from VA-ECMO.

*Trial registration* Retrospectively registered in UMIN-CTR (Reference No. R00006352).

**Supplementary Information:**

The online version contains supplementary material available at 10.1186/s40560-024-00761-z.

## Background

Combination therapy with veno-arterial extracorporeal membrane oxygenation (VA-ECMO) and Impella (ECPELLA) has been recently proposed for patients with cardiogenic shock (CS) requiring mechanical circulatory support (MCS). This method has been reported to reduce myocardial damage and improve prognosis by alleviating the increased left ventricular (LV) afterload caused by retrograde blood flow with VA-ECMO [1]. ECPELLA has been reported to reduce 30-day mortality by 20%–25% compared with VA-ECMO alone, but the 30-day mortality rate remains high (> 50%) in patients treated with ECPELLA [1, 2]. Therefore, we believe that reducing the mortality of patients treated with ECPELLA is the future challenge that needs to be overcome to improve the prognosis of patients with CS.

Inhaled nitric oxide (iNO) is taken up transalveolarly into the pulmonary capillaries, making it possible to efficiently improve respiratory and right ventricular (RV) function without any hypotensive side effects in the systemic circulation. This is possible due to selective vasodilation of the pulmonary arteries by iNO and improvements in ventilatory blood flow ratio and intrapulmonary shunting. [3]

iNO has been reported to improve pediatric pulmonary artery hypertension (PAH) and perioperative PAH in adult cardiac surgery [4, 5]. iNO has been used in pediatric and adult cardiac surgery for over three decades, but few reports have evaluated its efficacy in patients with CS requiring MCS.

Early weaning from VA-ECMO may be the first step toward reducing the mortality of ECPELLA patients. iNO therapy improves RV function by decreasing pulmonary arterial resistance, resulting in an increased Impella flow rate, which may facilitate early weaning from VA-ECMO and improve survival. The purpose of this study is to investigate the prognostic and hemodynamic impact of iNO therapy in ECPELLA patients.

## Methods

### Study population and procedure

This study was a single-center retrospective observational study and included 48 consecutive patients supported with ECPELLA for CS due to acute myocardial infarction (AMI) and non-compensated heart failure at Kindai University Hospital from September 2019 to March 2024. The purpose of iNO introduction and the doses used were left to the discretion of the attending physicians. Because iNO has been approved in Japan for improving perioperative PAH during cardiac surgery, it was introduced when the physician considered the clinical condition to be equivalent in the present study. VA-ECMO circuit blood flow and Impella support levels were adjusted at the discretion of each physician. Temperature control therapy for cardiopulmonary arrest (CPA) patients had performed by a specific protocol in our hospital that a constant temperature between 35 °C and 36 °C should be maintained for at least 24 h after achieving target temperature [6]. The present study was conducted in accordance with the Declaration of Helsinki and was approved by the Kindai University Hospital Institutional Review Board (Reference No. R06-091), and an opt-out method was used to obtain consent from patients.

### Clinical endpoints and measurements

The primary endpoint was 30-day all-cause mortality. The secondary endpoints were VA-ECMO withdrawal rate and duration between patients with and without iNO therapy, as well as changes in hemodynamic- and MCS-related parameters after the initiation of iNO therapy. The hemodynamic parameters included arterial pressure, cardiac output, cardiac index, pulmonary artery (PA) pressure, and pulmonary artery pulsatility index (PAPi). The MCS-related parameters included flow and rotation per minute or P support levels with VA-ECMO and Impella before and after iNO initiation. Changes in the PAPi and Impella parameters were evaluated before and at 2, 6, 12, and 24 h after iNO initiation. Similar parameters were evaluated in patients with and without iNO at the same time points from ECPELLA insertion.

The PAPi was calculated according to the following formula [7]: PAPi = (PA systolic pressure − PA diastolic pressure) ÷ right atrial pressure. We reviewed the medical records to identify patients’ characteristics. Blood tests (creatine kinase [CK]-MB, brain natriuretic peptide [BNP], estimated glomerular filtration rate, lactate, pH, bicarbonate, methemoglobin), echocardiography (LV ejection fraction [LVEF]), presence of iNO, duration of iNO, maximum dose of iNO, cause of CS, and 30-day all-cause mortality were also investigated. All PA catheter data, including pressure data and cardiac output measured by the thermodilution method, were obtained at rest during ongoing treatment without drug testing or exercise stress testing.

### Statistical analysis

Continuous variables are presented as the mean ± standard deviation or median with range [interquartile range], and categorical variables are presented as frequency (percentage). The Wilcoxon rank-sum test was used to compare the continuous variables, while Fisher’s exact test was used to compare the categorical variables between patients with and without iNO therapy. To compare variables between different time points in the same individual, the paired t-test was used. Multivariate analysis of variance was used to compare the parameters over time between the two groups. The Kaplan–Meier method was used to estimate cumulative incidences, and the log-rank test was used to compare the cumulative incidences between patients with and without iNO therapy. To eliminate bias prior to iNO use, additional landmark methods were used. To assess the association between iNO therapy and 30-day mortality, a multivariate logistic regression analysis was performed to account for the following potential confounders identified based on clinical knowledge and data available from previous studies [8]; lactate levels, mean arterial pressure (MAP) < 60 mmHg on admission. The odds ratio (OR) and 95% confidence interval (CI) were calculated. Of all the analyses, only cardiac output had missing data because it was sometimes impossible to calculate while using VA-ECMO. Cardiac output was analyzed using only the data obtained without using the data imputation method because it was not the main purpose of the analysis. Statistical significance was set at P < 0.05. All statistical analyses were performed using JMP Pro 16.2 (SAS Institute).

## Results

### Patient characteristics

Of the 48 ECPELLA patients, 25 were treated with iNO and 23 were without iNO. The patients’ characteristics are presented in Table 1. The mean age of the patients was 68.8 ± 13.4 years, and 36 patients were male (75.0%). AMI was the most common cause of CS (23 patients [47.9%]), of which 13 patients (27.1%) had ST-segment elevation myocardial infarction. In terms of the laboratory results of the enrolled patients, the median peak CK-MB was 251 [65–629] U/L, median BNP was 697 [126–1763] pg/mL, and median LVEF was 25% [15%–30%] on echocardiography. There were no significant differences between the two groups in age, sex, BMI, cause of CS, laboratory data, and LVEF on echocardiography, but hypertension and smoking history were significantly more common in patients without iNO therapy than in those with iNO therapy.

**Table 1:** Patients’ baseline characteristics

	All cases (n = 48)	With iNO therapy (n = 25)	Without iNO therapy (n = 23)	P value
Baseline				
Age, years	68.8 ± 13.4	69.2 ± 14.3	68.4 ± 12.6	0.85
Male sex, *n* (%)	36 (75.0)	21 (84.0)	15 (65.2)	0.13
BMI	22.6 (19.5–26.5)	20.2 (17.5–25.1)	24.2 (22.3–27.1)	0.26
Hypertension, *n* (%)	30 (62.5)	19 (76.0)	11 (47.8)	0.04
Diabetes, *n* (%)	23 (47.9)	13 (52.0)	10 (43.5)	0.55
Dyslipidemia, *n* (%)	24 (50.0)	13 (52.0)	11 (47.8)	0.77
Current or past smoker, *n* (%)	26 (54.2)	17 (68.0)	9 (39.1)	0.04
CPA, n (%)	24 (50.0)	13 (52.0)	11 (47.8)	0.77
MVD, n (%)	17 (35.4)	8 (32.0)	9 (39.1)	0.61
MAP < 60 mmHg, *n* (%)	29 (60.4)	14 (56.0)	15 (65.2)	0.51
Drug therapy on pulmonary circulation, *n* (%)				
Dobutamine	44 (91.7)	24 (96.0)	20 (87.0)	0.25
Adrenaline	12 (25.0)	5 (20.0)	7 (30.4)	0.40
Milrinone	6 (12.5)	4 (16.0)	2 (8.7)	0.44
Cause of CS, *n* (%)				
AMI	27 (56.3)	13 (52.0)	14 (60.9)	0.54
-STEMI	14 (29.2)	5 (20.0)	9 (39.1)	0.14
-NSTEMI	13 (27.1)	8 (32.0)	5 (21.7)	0.42
AHF with low EF	13 (27.1)	9 (36.0)	4 (17.4)	0.14
Acute myocarditis	6 (12.5)	3 (12.0)	3 (13.0)	0.91
VT/VF	2 (4.2)	0 (0)	2 (8.7)	0.08
Laboratory and echo data on admission				
Peak CK-MB (U/L)	251 (65–629)	140 (57–569)	339 (63–731)	0.18
BNP (pg/mL)	697 (126–1763)	909 (210–1952)	356 (98–1302)	0.89
Lactate (mg/dL)	73 (22–131)	48 (21–114)	121 (46–138)	0.07
pH	7.35 (7.14–7.44)	7.42 (7.18–7.48)	7.28 (7.06–7.39)	0.06
HCO_3_^−^ (mmol/L)	18.9 (14.2–20.9)	19.4 (14.8–21.2)	18.3 (13.7–20.9)	0.66
eGFR (mL/min/1.73m2)	46.0 (30.5–56.8)	40.0 (28.5–55.9)	48.0 (34.0–58.0)	0.60
LVEF (%)	25 (15–30)	25 (18–30)	26 (15–40)	0.40

*iNO* inhaled nitric oxide, *BMI* body mass index, *CPA* cardiopulmonary arrest, *MVD* multivessel coronary artery disease, *MAP* mean arterial pressure, *CS* cardiogenic shock, *AMI* acute myocardial infarction, *(N)STEMI* (Non-)ST-segment elevation myocardial infarction, *AHF* acute heart failure, *EF* ejection fraction, *VT/VF* ventricular tachycardia/fibrillation, *BNP* brain natriuretic peptide, *eGFR* estimated glomerular filtration rate, *LVEF* left ventricular ejection fraction

### Clinical outcomes

The clinical outcomes of the patients are shown in Table 2. There were no significant differences in complications between patients with and without iNO therapy, but the 30-day mortality rate was significantly lower in patients with iNO (24% vs. 73.9%, P = 0.0004). Cardiac death was the most common cause of death (17 patients [35.4%]), which was significantly more common in patients without iNO. Conversely, multiple organ failure was significantly more common in patients with iNO than in patients without iNO. Other causes of death were not significantly different between patients with and without iNO.

**Table 2:** Clinical outcomes

	All cases (*n* = 48)	With iNO therapy (*n* = 25)	Without iNO therapy (*n* = 23)	P value
30-day mortality, *n* (%)	23 (47.9)	6 (24.0)	17 (73.9)	0.0004
Cause of death, *n* (%)				
Cardiac death	17 (35.4)	2 (8.0)	15 (65.2)	< 0.0001
Cerebral complications	1 (2.1)	0 (0)	1 (4.3)	0.22
Hemorrhagic complications	1 (2.1)	0 (0)	1 (4.3)	0.22
MOF	3 (6.3)	3 (12.0)	0 (0)	0.04
Sepsis	1 (2.1)	1 (4.0)	0 (0)	0.25
Death during MCS, *n* (%)				
During ECPELLA support	15 (31.3)	3 (12.0)	12 (52.3)	0.0021
Cardiac death	11 (22.9)	1 (4.0)	10 (43.5)	0.10
Hemorrhagic complications	1 (2.1)	0 (0)	1 (4.3)	0.50
Sepsis	3 (6.3)	2 (8.0)	1 (4.3)	0.38
During Impella support	6 (12.5)	1 (4.0)	5 (21.7)	0.05
Cardiac death	4 (8.3)	0 (0)	4 (17.4)	0.0016
Cerebral complications	1 (2.1)	0 (0)	1 (4.3)	0.13
Sepsis	1 (2.1)	1 (4.0)	0 (0)	0.36
Complications, *n* (%)				
Hemolysis	1 (2.1)	1 (4.0)	0 (0)	0.25
Major bleeding	21 (43.8)	12 (48.0)	9 (39.1)	0.54
Peripheral ischemia	3 (6.3)	1 (4.0)	2 (8.7)	0.50
Stroke	7 (14.6)	3 (12.0)	4 (17.4)	0.60
Thrombosis	0 (0)	0 (0)	0 (0)	-
Hypoxic encephalopathy	15 (31.3)	5 (20.0)	10 (43.5)	0.08
Methemoglobinemia	0 (0)	0 (0)	0 (0)	-

*iNO* inhaled nitric oxide, *MOF* multiple organ failure, *MCS* mechanical circulatory support, *ECPELLA* Veno-arterial extracorporeal membrane oxygenation with Impella

**Fig. 1:**
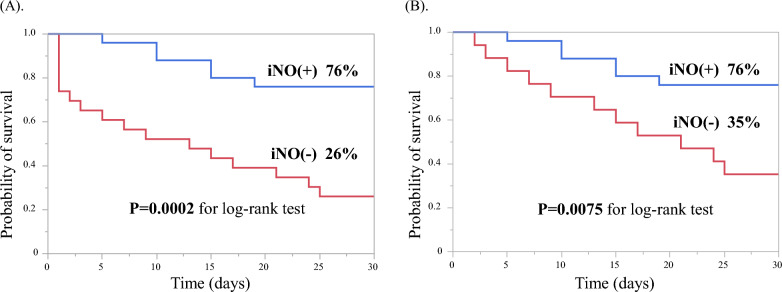
Kaplan–Meier survival curves. **A** Kaplan–Meier survival curves of patients with ECPELLA with and without iNO therapy. The Kaplan–Meier survival analysis demonstrated that the 30-day survival rate was significantly higher in patients with iNO therapy than in patients without iNO therapy (76% vs. 26%, log-rank P = 0.0002). **B** Landmark analysis after iNO therapy. Similar results were obtained in the landmark analysis after iNO initiation, with a significantly higher 30-day survival rate in patients with iNO therapy than in patients without iNO therapy (76% vs. 35%, log-rank P = 0.0075). *iNO* inhaled nitric oxide, *ECPELLA* veno-arterial extracorporeal membrane oxygenation with Impella

The Kaplan–Meier survival analysis demonstrated that the 30-day survival rate was significantly higher in patients with iNO than in those without iNO (76% vs. 26%, log-rank P = 0.0002, Fig. 1A). Similar results were obtained in the landmark analysis after iNO initiation, with a significantly higher 30-day survival rate in patients with iNO than in those without iNO (76% vs. 35%, log-rank P = 0.0075, Fig. 1B).

**Fig. 2:**
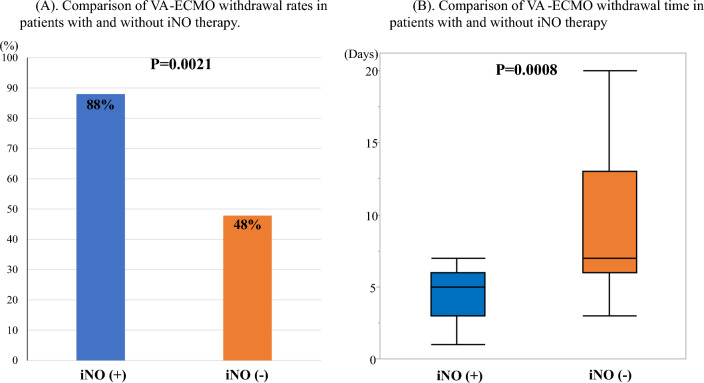
The data about VA-ECMO withdrawal. **A** Comparison of the rate of VA-ECMO withdrawal between patients with and without iNO therapy. Patients with iNO therapy had a significantly higher VA-ECMO withdrawal rate than those without iNO therapy (88% vs. 48%, P = 0.0021). **B** Comparison of the time of VA-ECMO withdrawal between patients with and without iNO therapy. Patients with iNO therapy had a shorter time to VA-ECMO withdrawal than those without iNO therapy (5 [3–6] days vs. 7 [6–13] days, P = 0.0008). *iNO* inhaled nitric oxide, *VA-ECMO* veno-arterial extracorporeal membrane oxygenation

Patients with iNO had a significantly higher VA-ECMO withdrawal rate (88% vs. 48%, P = 0.0021, Fig. 2A) and a shorter time to VA-ECMO withdrawal (5 [3–6] days vs. 7 [6–13] days, P = 0.0008, Fig. 2B) than patients without iNO. Impella withdrawal rates were significantly higher in patients with iNO than in those without iNO (88% vs. 30.4%, P < 0.01), but there was no significant difference in time to withdrawal (6.9 [5.3 – 9.8] days vs. 6.3 [4.2 – 12.0] days, P = 0.57). Twenty-one deaths occurred during MCS, including 15 during ECPELLA and 6 during Impella. The main causes of death during MCS are shown in Table 2.

A comparison of patient background with and without 30-day mortality is shown in Supplementary Table 1. Patients who died within 30 days had significantly lower MAP, pH and proportion of acute myocarditis, higher proportion of CPA on arrival, and less treated with iNO. Multivariate logistic regression analysis showed that iNO therapy was independent predictor for 30-day mortality (iNO; OR 0.10, 95% CI, 0.02—0.46, P < 0.01, MAP < 60 mmHg; OR 5.51, 95% CI 0.93—32.8, P = 0.06, lactate levels; OR 1.00, 95% CI 0.99—1.01, P < 0.99).

### Hemodynamic- and MCS-related parameters before and after iNO initiation

The details of hemodynamic- and MCS-related parameters are shown in Table 3. The mean number of days to iNO initiation from MCS insertion was 1.5 ± 1.3 days, and the mean duration of iNO therapy was 13.1 ± 8.1 days, with a maximum dose of 21.2 ± 6.0 ppm. There was no significant difference in the time from hospital admission or presentation of CS to the introduction of VA-ECMO (1.3 h (0.6–10.5) vs. 3.0 h (0.5–7.0), P = 0.99) and Impella (4.4 h (2.5–27.0) vs. 5.5 h (2.5–39.0), P = 0.18) between patients with iNO and without iNO. The majority of ECPELLA patients used Impella CP (87.5%). There were no significant differences between the two groups in MCS- and hemodynamic-related parameters before and 24 h after iNO initiation, including VA-ECMO and Impella flow, MAP, and PAPi.

**Table 3:** MCS flow, and changes in hemodynamic and oxygenation parameters before and after iNO therapy initiation

	All cases (*n* = 48)	With iNO therapy (*n* = 25)	Without iNO therapy (*n* = 23)	P value
iNO therapy				
Days to iNO initiation (days)	–	1.5 ± 1.3	–	–
Duration of iNO (days)	–	13.1 ± 8.1	–	–
Maximum dose of iNO (ppm)	–	21.2 ± 6.0	–	–
MCS				
VA-ECMO flow (L/min)				
-Pre	2.1 (2.3–3.6)	3.1 (2.0–3.3)	3.4 (2.7–3.9)	0.21
-Post 24 h	2.9 (2.3–3.6)	2.7 (2.2–3.3)	3.2 (2.8–3.8)	0.23
Impella CP, n (%)	42 (87.5)	23 (92.0)	19 (82.6)	0.25
Impella P levels				
-Pre	4.0 (2.0–5.8)	4.0 (2.5–6.0)	3.0 (2.0–5.0)	0.32
-Post 24 h	4.0 (2.0–6.0)	4.0 (2.0–6.0)	4.0 (2.0–5.5)	0.77
Impella flow (L/min)				
-Pre	1.7 (1.3–2.3)	1.5 (1.3–2.3)	1.9 (1.3–2.3)	0.80
-Post 24 h	2.0 (1.4–2.6)	2.0 (1.4–2.9)	2.1 (1.5–2.4)	0.46
Hemodynamics				
MAP (mmHg)				
-Pre	77 (66–86)	78 (71–89)	72 (50–85)	0.08
-Post 24 h	75 (66–88)	79 (66–88)	73 (64–89)	0.53
CO (L/min)				
-Pre^*a^	2.3 (1.5–3.2)	2.3 (1.3–3.8)	2.2 (1.8–3.2)	0.69
-Post 24 h^*b^	2.7 (2.0–3.5)	3.2 (1.8–3.8)	2.6 (1.9–3.2)	0.65
Mean PA pressure (mmHg)				
-Pre	18 (15–24)	18 (16–24)	17 (12–23)	0.35
-Post 24 h	19 (14–21)	19 (15–22)	15 (13–21)	0.25
RA pressure (mmHg)				
-Pre	10 (7–13)	11 (8–13)	9 (6–15)	0.95
-Post 24 h	9 (6–12)	9 (7–12)	9 (4–13)	0.96
PAPi score				
-Pre	1.1 (0.6–1.7)	1.0 (0.7–1.5)	1.6 (0.5–2.1)	0.14
-Post 24 h	1.5 (1.0–2.3)	1.5 (1.0–2.0)	1.6 (0.9–2.7)	0.65
Oxygenation				
P/F ratio (patient)				
-Pre	329 (216–459)	312 (189–456)	340 (250–466)	0.86
-Post 24 h	308 (196–419)	370 (199–408)	255 (190–428)	0.77
P/F ratio (VA-ECMO)				
-Pre	433 (336–502)	467 (362–547)	369 (329–456)	0.08
-Post 24 h	452 (360–538)	473 (386–563)	410 (243–535)	0.21

*MCS* mechanical circulatory support, *VA-ECMO* veno-arterial extracorporeal oxygenation, *MAP* mean arterial pressure, *CO* cardiac output, *PA* pulmonary artery, *RA* right atrial, *PAPi* pulmonary artery pulsatility index, *P/F ratio* PaO_2_/FiO_2_ ratio, *iNO* inhaled nitric oxide

^*^^a^: data were available for 12 patients with iNO therapy and 8 patients without iNO therapy

^*^^b^: data were available for 13 patients with iNO therapy and 7 patients without iNO therapy

**Fig. 3:**
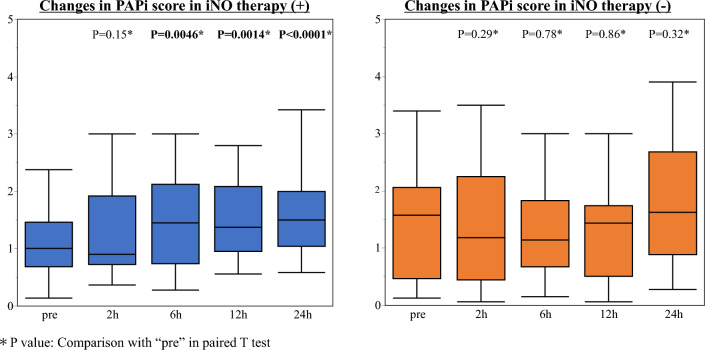
Changes in hemodynamic parameters before and after iNO therapy. The changes in PAPi from before to 24 h after iNO introduction in patients with and without iNO therapy. The PAPi in patients with iNO increased over time and was significantly higher after 6 h than before iNO initiation. There was no significant change in the PAPi over time in patients without iNO therapy. These results imply that iNO therapy in ECPELLA patients improves RV function. *ECPELLA* veno-arterial extracorporeal membrane oxygenation with Impella, *iNO* inhaled nitric oxide, *PAPi* pulmonary artery pulsatility index

**Fig. 4:**
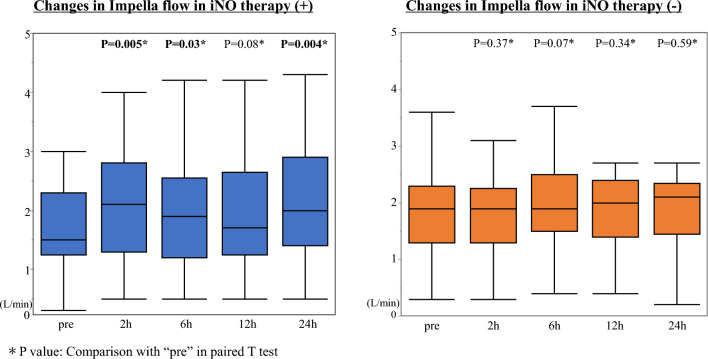
Changes in Impella flow before and after iNO therapy. Impella flow in patients with iNO significantly increased after iNO initiation in 24 h, while there was no change in patients without iNO, and also no change of Impella support levels in two groups. *iNO* inhaled nitric oxide

The changes in PAPi from before to 24 h after iNO introduction between patients with and without iNO are shown in Fig. 3. The changes in Impella flow and Impella P levels over time are shown in Fig. 4. The PAPi and Impella flow showed a significant increase after iNO initiation, while there was no change in patients without iNO. In addition, a comparison of the change over time between the two groups for Impella flow suggested a significant increase in patients with iNO (P = 0.04). Hemodynamic- and MCS-related parameters before and after withdrawal of VA-ECMO and Impella are shown in Supplementary Table 2. The PaO2/FiO2 (P/F) ratio was not significantly different between the two groups, either before or after the introduction of iNO therapy (Table 3).

## Discussion

### Major findings

The key finding of the present study is that 30-day mortality in patients with CS supported by ECPELLA was improved by adjuvant iNO therapy. There have been only a few case reports on the efficacy of iNO therapy in patients with ECPELLA-supported CS, and this is the first observational study to determine the prognostic and hemodynamic impact of iNO in consecutive ECPELLA patients. The prognostic impact of iNO use was attributed to the fact that patients who used iNO had significantly higher VA-ECMO withdrawal rates and shorter withdrawal times, which proved to be an effect of improved RV function, increased Impella flow, and improved respiratory function. The present study demonstrated that adjuvant iNO therapy for patients with CS supported by ECPELLA may be a useful therapeutic option.

### Hemodynamic features of patients supported by ECPELLA or VA-ECMO

VA-ECMO support is a lifesaving tool in cardiovascular care, and it provides excellent hemodynamic assistance for numerous indications, such as CS or periprocedural support in patients with acute heart failure [9]. The use of VA-ECMO support has increased during the past 10 years. However, the mortality rate of these patients remains high, and the results of a recent multicenter randomized controlled trial showed no superiority of VA-ECMO use in terms of 30-day mortality in patients of AMI with CS [10]. This may be because the potential benefits of hemodynamic support are offset by increased LV afterload due to retrograde flow with VA-ECMO, as well as device-related local and systemic complications, such as bleeding, stroke, limb ischemia, and hemolysis [1, 10, 11]. Several reports have evaluated the efficacy of mechanical unloading of LV afterload, demonstrating that LV unloading with Impella support under VA-ECMO is associated with lower short-term mortality, despite increased complications [1, 2, 11–13]. However, J-PVAD, a registry study of Impella in Japan, reported that the 30-day survival rate of patients with AMI with CS was 80.9% with Impella alone, but it remained as low as 45.7% with ECPELLA, indicating that clinical problems still remain [13]. To further improve the prognosis of patients with ECPELLA, it is important to prevent complications, such as bleeding, thrombosis, and infection, and early weaning from VA-ECMO is considered to be of the highest priority. We therefore focused our study on the importance of improving RV function and oxygenation to achieve early weaning from VA-ECMO and predominant assistance with Impella flow. In fact, previous reports have shown that the in-hospital mortality rate of patients with RV failure is as high as 70%–75% [14–16], indicating that RV function is strongly related to mortality, and that improving RV and respiratory function is key to improving survival in ECPELLA patients.

### Favorable effects of iNO therapy

iNO selectively vasodilates the pulmonary vessels via cyclic guanosine monophosphate production in pulmonary artery smooth muscle cells, and its ability to decrease pulmonary artery pressure and vascular resistance, as well as improve gas exchange, has led to its use in the management of RV failure. Nitric oxide is scavenged by hemoglobin upon diffusing into the blood and is thereby rapidly inactivated; therefore, the vasodilatory effect of iNO is limited to the lungs, thus preventing systemic hypotension. [17, 18]

Serious side effects of iNO are rare, with methemoglobinemia being considered as almost the only serious side effect of iNO. The normal level of methemoglobin is < 1%, and an increase to 10% results in cyanosis [19], but there have been few cases of life-threatening methemoglobinemia induced by iNO. [20]

At present, the only indication for which iNO therapy is approved by the Food and Drug Administration is the treatment of persistent PAH of the newborn [21]. Along with this indication, iNO has been considered as a potential treatment for other diseases, including PAH in adults [22] and acute respiratory distress syndrome [19]. Moreover, recent studies have shown that the use of iNO prevented hemolysis-induced vasoconstriction [23], decreased ischemia/reperfusion injury [24], and prevented renal failure associated with cardiopulmonary bypass [25]. In addition, the hemodynamic effects of iNO therapy in patients with RV myocardial infarction and CS were reported in 2004, and it was shown that iNO significantly decreased mean right arterial pressure, mean pulmonary artery pressure, and pulmonary vascular resistance, as well as increasing cardiac and stroke volume indices. Furthermore, a recent report examining changes in hemodynamic indices after the introduction of iNO in 11 ECPELLA patients observed a significant increase in the PAPi and Impella flow after the introduction of iNO [26]. However, the clinical efficacy of iNO in patients with ECPELLA remained unclear due to the lack of a control group. Moreover, it is possible that hemodynamic improvement occurred as part of the natural course of cardiac recovery. Furthermore, the survival rate and VA-ECMO withdrawal rate were not compared between patients with and without iNO therapy.

Therefore, in the present retrospective study, we included a control group of patients without iNO therapy to determine whether iNO contributes to hemodynamic improvement, particularly in terms of RV function; increases Impella flow; and clinically influences VA-ECMO withdrawal and 30-day mortality. The results of this study showed a significant improvement in the PAPi over time and a trend toward increased Impella flow in patients with iNO, while the same significant changes were not observed in patients without iNO. Although iNO therapy risks worsening pulmonary arterial wedge pressure by increasing pulmonary venous return in patients with severe LV systolic dysfunction, its use with Impella support may have favorable results in terms of increasing cardiac output without increasing pulmonary arterial wedge pressure.

Furthermore, previous studies on hemolysis and RV failure in patients with Impella have reported that decreased LV preload due to impaired RV function may reduce intra-device LV blood flow, increasing shear stress and promoting hemolysis [27]. Therefore, increasing LV preload with iNO may have a favorable effect in terms of preventing hemolysis. Theoretically, we expected oxygenation to improve before and after the introduction of iNO, but no significant changes were observed, possibly because the greater impact of strong oxygenation under the VA-ECMO-dominated assisted circulation drowned out any improvement in oxygenation by iNO.

In the present study, the favorable hemodynamic effects may have led to significantly improved VA-ECMO withdrawal rates and withdrawal durations, as well as significantly improved 30-day survival rates. Conversely, methemoglobinemia, thought to be the only complication of iNO, was not observed in this study. Our findings suggest that early adjunctive iNO therapy in patients with ECPELLA is safe and may be useful to stabilize hemodynamics and improve clinical outcomes.

## Limitations

This study has some limitations that should be considered. Patients diagnosed with hypoxic encephalopathy may have influenced the decisions for further invasive additional treatment, and may have affected 30-day mortality. In particular, as the data were obtained from a small number of patients at a single center and a retrospective study design was used, the possibility of selection bias cannot be excluded. Therefore, prospective controlled trials with a unified protocol are needed in the future to clarify our observations.

## Conclusion

iNO therapy in patients with CS requiring ECPELLA was associated with short-term prognosis by improving RV function and facilitating weaning from VA-ECMO.

## Supplementary Information

Additional file 1: Table 1. Factors related to 30-day mortality. Table 2. Changes in hemodynamic parameters before and after withdrawal of VA-ECMO and Impella.

## Data Availability

The data underlying the article are available within the article and its online supplementary material.

## Abbreviations

AMI: Acute myocardial infarction
CI: Confidence interval
CPA: Cardiopulmonary arrest
CS: Cardiogenic shock
ECPELLA: Veno-arterial extracorporeal membrane oxygenation combined with Impella
iNO: Inhaled nitric oxide
LV: Left ventricular
LVEF: Left ventricular ejection fraction
MAP: Mean arterial pressure
MCS: Mechanical circulatory support
OR: Odds ratio
PA: Pulmonary artery
PAH: Pulmonary arterial hypertension
PAPi: Pulmonary artery pulsatility index
RV: Right ventricular
VA-ECMO: Veno-arterial extracorporeal membrane oxygenation
