# Supplementary material for: Impact of inhaled nitric oxide therapy in patients with cardiogenic shock treated with veno-arterial extracorporeal membrane oxygenation combined with Impella: a retrospective cohort study
Source: J Intensive Care. 2024 Nov 18;12:49. doi: 10.1186/s40560-024-00761-z (PMC11572410; doi:10.1186/s40560-024-00761-z)
Supplement: Supplementary file 1 — Additional file 1: Table 1. Factors related to 30-day mortality. Table 2. Changes in hemodynamic parameters before and after withdrawal of VA-ECMO and Impella. [file 40560_2024_761_MOESM1_ESM.docx]

Supplementary Table 1. Factors related to 30-day mortality.

|  | **30-day death (+)**  **N=23** | **30-day death (-)**  **N=25** | **P value** |
| --- | --- | --- | --- |
| **Baseline** | | | |
| Age (years) | 69.4±12.0 | 68.2±14.8 | 0.75 |
| Male sex, n (%) | 17 (73.9) | 19 (76.0) | 0.87 |
| BMI | 24.8 (20.0-26.9) | 21.9 (17.8-24.0) | 0.08 |
| CPA, n (%) | 15 (65.2) | 9 (36.0) | 0.04 |
| MVD, n (%) | 9 (36.0) | 8 (34.8) | 0.93 |
| **Cause of CS** | | | |
| STEMI, n (%) | 8 (34.8) | 6 (24.0) | 0.41 |
| AHF with low EF, n (%) | 7 (30.4) | 6 (24.0) | 0.62 |
| Acute myocarditis, n (%) | 0 (0) | 6 (24.0) | <0.01 |
| VT/VF, n (%) | 1 (4.4) | 1 (4.0) | 0.95 |
| **Laboratory and echo data on admission** | | | |
| Peak CK-MB (ng/mL) | 457 (28-680) | 140 (68-607) | 0.78 |
| BNP (pg/mL) | 297 (41-909) | 1123 (253-1900) | 0.91 |
| Lactate (mg/dL) | 102 (31-137) | 57 (20-115) | 0.12 |
| pH | 7.21 (7.06-7.41) | 7.42 (7.30-7.45) | 0.05 |
| eGFR (mL/min/1.73m^2)^ | 48 (32-56) | 40 (25-57) | 0.95 |
| LVEF (%) | 26 (15-35) | 20 (16-30) | 0.56 |
| **Hemodynamics before introduction of iNO** | | | |
| MAP (mmHg) | 72 (50-85) | 78 (72-89) | 0.0088 |
| PAPi score | 1.1 (0.5-1.7) | 1.1 (0.7-1.7) | 0.96 |
| **iNO therapy** | | | |
| With iNO therapy, n (%) | 6 (26.1) | 19 (76.0) | 0.0004 |

BMI: body mass index; CPA: cardiopulmonary arrest; MVD: multivessel coronary artery disease; CS: cardiogenic shock; STEMI: ST-segment elevation myocardial infarction; AHF: acute heart failure; EF: ejection fraction; BNP: brain natriuretic peptide; eGFR: estimated glomerular filtration rate; LVEF: left ventricular ejection fraction; MAP: mean arterial pressure; PAPi: pulmonary artery pulsatility index; iNO: inhaled nitric oxide.

Supplementary Table 2. Changes in hemodynamic parameters before and after withdrawal of VA-ECMO and Impella

|  | **All cases**  **(n=48)** | **With iNO therapy**  **(n=25)** | **Without iNO therapy**  **(n=23)** | **P value** |
| --- | --- | --- | --- | --- |
| **Before withdrawal of VA-ECMO** | | | | |
| CO (L/min) | 3.3 (2.3-4.2) | 3.1 (2.2-3.8) | 3.9 (2.4-4.7) | 0.30 |
| PAPi score | 1.5 (1.1-3.0) | 2.1 (1.0-3.1) | 1.3 (1.1-2.4) | 0.30 |
| Impella flow (L/min) | 2.7 (2.0-3.1) | 2.7 (2.0-3.0) | 2.9 (2.1-3.9) | 0.20 |
| Impella P levels | 6.0 (5.0-7.0) | 6.0 (5.0-7.0) | 6.5 (4.8-7) | 0.97 |
| **After withdrawal of VA-ECMO** | | | | |
| CO (L/min) | 4.2 (3.4-5.3) | 4.1 (3.2-5.2) | 4.2 (3.4-5.4) | 0.44 |
| PAPi score | 2.1 (1.2-3.0) | 2.1 (1.0-3.2) | 2.0 (1.2-2.8) | 0.41 |
| Impella flow (L/min) | 3.0 (2.4-3.6) | 3.0 (2.4-3.5) | 3.0 (2.5-4.5) | 0.39 |
| Impella P levels | 6.0 (4.0-7.0) | 6.0 (4.0-7.0) | 6.5 (3.8-8.0) | 0.62 |
| **Before withdrawal of Impella** | | | | |
| CO (L/min) | 4.6 (3.5-5.4) | 4.4 (3.6-5.0) | 5.2 (2.6-6.3) | 0.82 |
| PAPi score | 2.0 (0.9-2.9) | 2.1 (0.9-3.1) | 1.4 (0.9-2.2) | 0.31 |
| **After withdrawal of Impella** | | | | |
| CO (L/min) | 4.1 (3.3-4.9) | 4.0 (3.3-5.0) | 4.7 (2.8-4.9) | 0.80 |
| PAPi score | 1.4 (1.0-3.4) | 1.4 (1.0-3.6) | 1.0 (0.9-2.2) | 0.38 |

VA-ECMO: veno-arterial extracorporeal oxygenation; CO: cardiac output; PAPi: pulmonary artery pulsatility index.
